# Supplementary material for: Tetrazene–Characterization of Its Polymorphs
Source: Molecules. 2021 Nov 24;26(23):7106. doi: 10.3390/molecules26237106 (PMC8658986; doi:10.3390/molecules26237106)
Supplement: Supplementary file 1 [file molecules-26-07106-s001.zip › molecules-1423780-supplementary.pdf]

molecules

Article

Tetrazene – Straightening the characterization of its polymorphism

Jan Ryšavý [1], Robert Matyáš [1,*], Zdeněk Jalový [1], Jaroslav Maixner [2], Aleš Růžička [3], Stanislav Brandejs [4], Jiří Nesveda [4]

[1] Institute of Energetic Materials, Faculty of Chemical Technology, University of Pardubice, Doubravice 41, 53210 Pardubice, Czech Republic.
[2] Central Laboratories, University of Chemistry and Technology Prague, Technicka 5, 16628 Prague 6, Czech Republic.
[3] Department of General and Inorganic Chemistry, Faculty of Chemical Technology, University of Pardubice, Studentska 573, 53210 Pardubice, Czech Republic.
[4] Sellier & Bellot a.s., Lidická 667, 25801 Vlašim, Czech Republic.
Correspondence: robert.matyas@upce.cz.

Contents

Indexed powder patterns....................page 2
Crystal data of tetrazene polymorphs....................page 3
FTIR spectra....................page 4
Raman spectra....................page 5

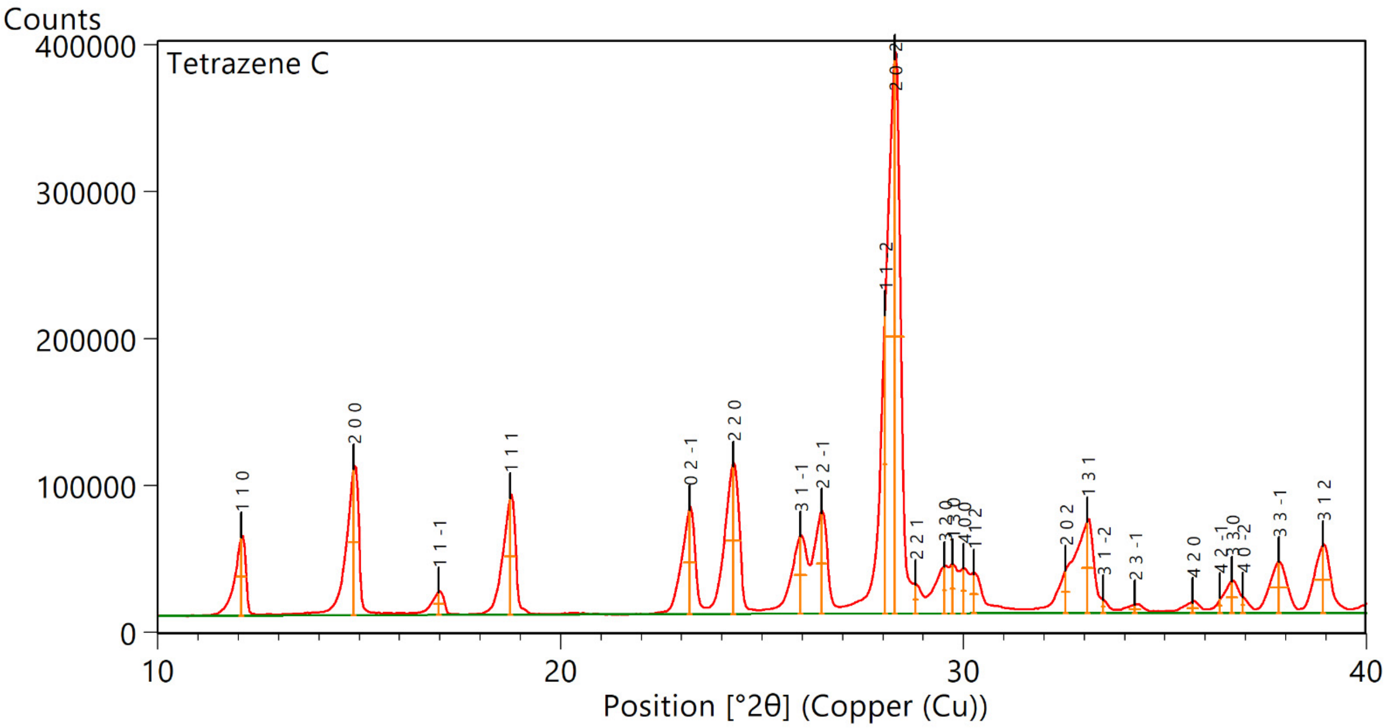

Figure S1 Indexed powder pattern of tetrazene C (range 10–40°2θ).

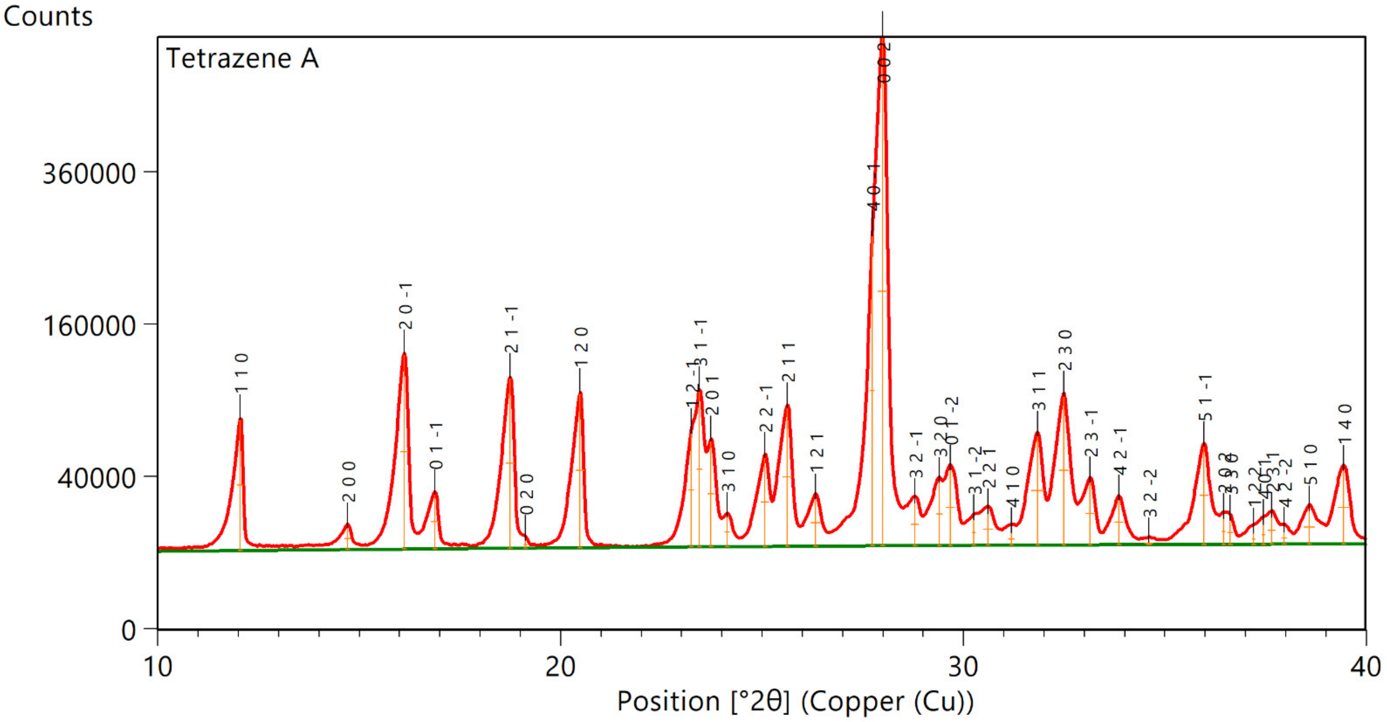

Figure S2 Indexed powder pattern of tetrazene A (range 10–40°2θ).

Table S1 Crystal data for tetrazene polymorphs A, B, C.

Compound	Formula	Space Group	Lattice Parameters			
			a (Å)	b (Å)	c (Å)	β (°)
Tetrazene A*	$C_2H_8N_{10}O$	*P*2$_1$/a	12.955(2)	9.295(1)	6.847(1)	111.54(1)
Tetrazene A**	$C_2H_8N_{10}O$	*P*2$_1$/a	12.958(3)	9.295(1)	6.845(2)	111.573(3)
Tetrazene B*	$C_2H_8N_{10}O$	*I*a	12.888(1)	9.332(1)	6.811(1)	112.47(1)
Tetrazene C**	$C_2H_8N_{10}O$	*C*c	12.070(5)	9.328(2)	6.815(2)	99.210(7)

*data presented by Duke [3], ** data from this study

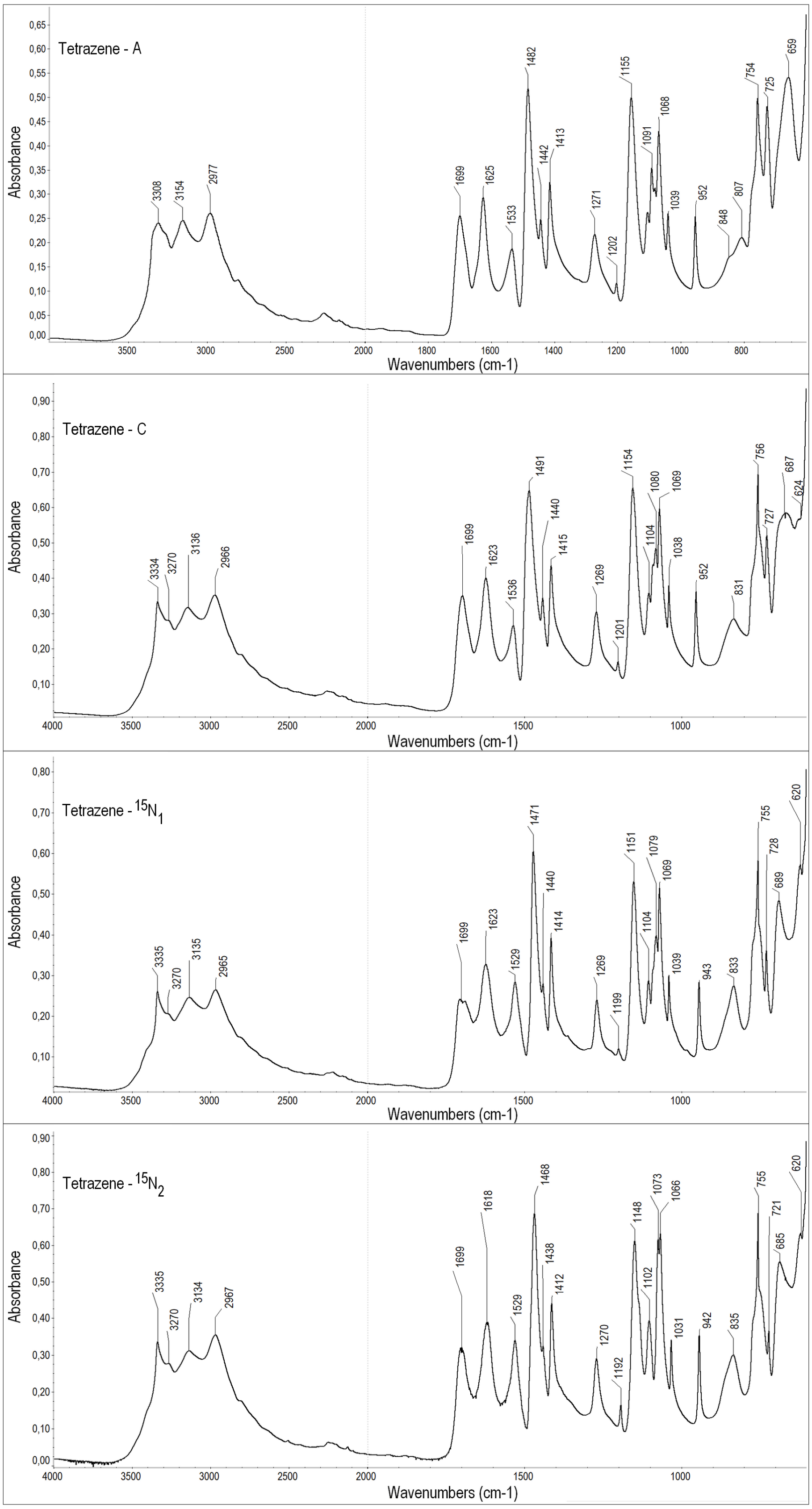

Figure S3 FTIR spectra of tetrazene (region 4000–600 cm^{-1}).

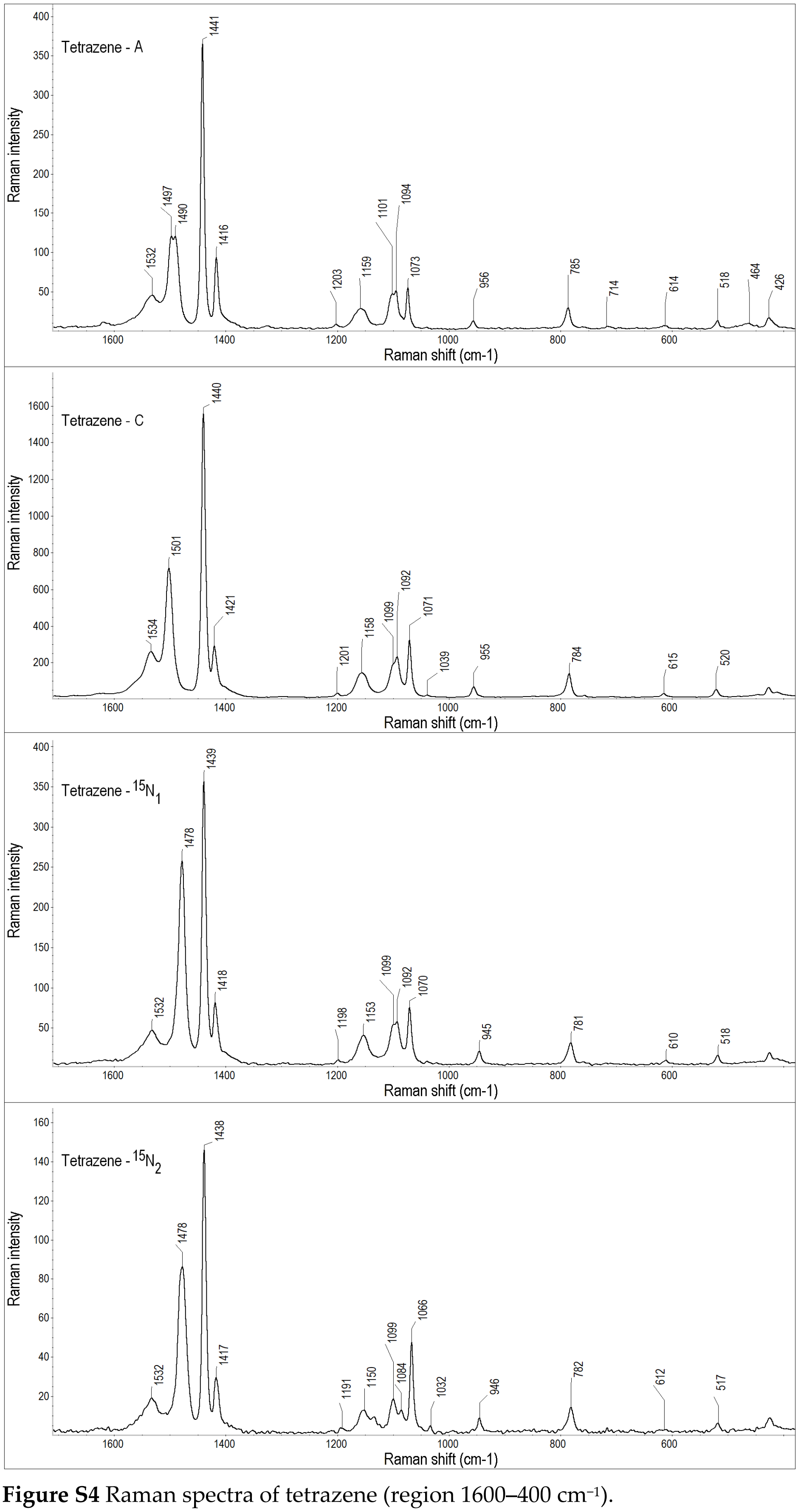

Figure S4 Raman spectra of tetrazene (region 1600–400 cm⁻¹).
